# Quantitative Molecular Detection of Putative Periodontal Pathogens in Clinically Healthy and Periodontally Diseased Subjects

**DOI:** 10.1371/journal.pone.0099244

**Published:** 2014-07-16

**Authors:** André Göhler, Adrian Hetzer, Birte Holtfreter, Marie Henrike Geisel, Carsten Oliver Schmidt, Ivo Steinmetz, Thomas Kocher

**Affiliations:** 1 Friedrich Loeffler Institute of Medical Microbiology, Ernst Moritz Arndt University, Greifswald, Germany; 2 Unit of Periodontology, Dental School, University Medicine, Ernst Moritz Arndt University, Greifswald, Germany; 3 Institute for Medical Informatics, Biometry and Epidemiology (IMIBE), University Hospital of Essen, University of Duisburg-Essen, Essen, Germany; 4 Section Methods in Community Medicine, Institute for Community Medicine, University Medicine Greifswald, Ernst Moritz Arndt University, Greifswald, Germany; University of Oklahoma Health Sciences Center, United States of America

## Abstract

Periodontitis is a multi-microbial oral infection with high prevalence among adults. Putative oral pathogens are commonly found in periodontally diseased individuals. However, these organisms can be also detected in the oral cavity of healthy subjects. This leads to the hypothesis, that alterations in the proportion of these organisms relative to the total amount of oral microorganisms, namely their abundance, rather than their simple presence might be important in the transition from health to disease. Therefore, we developed a quantitative molecular method to determine the abundance of various oral microorganisms and the portion of bacterial and archaeal nucleic acid relative to the total nucleic acid extracted from individual samples. We applied quantitative real-time PCRs targeting single-copy genes of periodontal bacteria and 16S-rRNA genes of *Bacteria* and *Archaea*. Testing tongue scrapings of 88 matched pairs of periodontally diseased and healthy subjects revealed a significantly higher abundance of *P. gingivalis* and a higher total bacterial abundance in diseased subjects. In fully adjusted models the risk of being periodontally diseased was significantly higher in subjects with high *P. gingivalis* and total bacterial abundance. Interestingly, we found that moderate abundances of *A. actinomycetemcomitans* were associated with reduced risk for periodontal disease compared to subjects with low abundances, whereas for high abundances, this protective effect leveled off. Moderate archaeal abundances were health associated compared to subjects with low abundances. In conclusion, our methodological approach unraveled associations of the oral flora with periodontal disease, which would have gone undetected if only qualitative data had been determined.

## Introduction

Periodontitis is a chronic multi-microbial infection characterized by destruction of the tooth supporting tissues which may result in tooth loss [1]. There is growing evidence that periodontal disease might predispose to various systemic diseases due to systemic circulation of inflammatory mediators and oral pathogens [2], [3]. Besides various *Bacteria*, *Archaea* seem to play an additional role in periodontal disease as indicated in recent reports [2]–[5]. However, the ecology of a number of oral prokaryotic species and their contribution in the pathogenesis of periodontal disease are still unclear [6]–[11]. Several studies indicated a fluctuating prevalence of *A. actinomycetemcomitans* among the studied subjects ([12] & references within). A recent literature-review by Hujoel and colleagues stated that no cohort studies supported *A. actinomycetemcomitans* as an etiologic agent of periodontal disease in adults [6]. Conflicting results in the literature might partly be explained by the wide range of methods used for the detection of putative periodontal pathogens.

Several qualitative and quantitative approaches [13]–[15] were previously applied to either detect or enumerate putative periodontal pathogenic microorganisms, including microbial culture methods, immunoassays [16], hybridization-based techniques such as whole-genomic checkerboard DNA-DNA hybridization [17] and DNA microarray technology [18]. Furthermore, endpoint and quantitative PCR (qPCR) analyses were conducted to analyze oral microorganisms (16–18). Compared with conventional detection methods, qPCR is superior for species detection and quantification because of its sensitivity, simplicity, and rapidness [19]. However, the majority of qPCR assays quantifying oral bacteria (for a comprehensive list see [20]) are based on the detection of the 16S ribosomal RNA gene whose copy number differs widely from one to 15 depending on the prokaryote [21] which might lead to inaccurate enumeration. Only few qPCR studies addressed this limitation by targeting single copy genes for direct and accurate quantification of oral bacteria [2], [8], [22]–[24]. To our knowledge, only the study by Yoshida et al. determined the abundance of a single bacterial species, namely *Aggregatibacter actinomycetemcomitans*, by qPCR targeting a single copy gene [22]. In this study, the fraction of *A. actinomycetemcomitans* from total bacteria in saliva and subgingival plaque was determined in ten subjects, but microbiological data were not correlated to periodontal status [22].

Therefore, we aimed to determine whether the portion of single prokaryotic oral species from total bacteria, namely their abundance, is associated with periodontal disease. qPCR assays based on single copy genes were used to quantify *Streptococcus sanguini*s, *Fusobacterium nucleatum, Porphyromonas gingivalis and A. actinomycetemcomitans* as tongue colonizers and representatives for different stages of oral biofilm formation [2], [21], [24], [25]. We further determined not only bacterial but also archaeal abundance as the portion of total nucleic acids isolated from single samples, by targeting 16S-rRNA genes. This methodological approach was validated in tongue samples of well-characterized periodontally diseased and periodontally healthy subjects.

## Materials and Methods

### Study population

Subjects were sampled from the five-year-follow-up of the Study of Health in Pomerania (SHIP). SHIP is a prospective cohort study in Pomerania, North-East Germany. Baseline examinations were held between 1997 and 2001 [26]. Briefly, a two-stage cluster sampling method adopted from the World Health Organization (WHO) MONICA Project in Augsburg, Germany [27] was used. From the entire population of 212,157 inhabitants, 7008 adults aged 20–79 years with German citizenship and main residency within the target region were randomly selected. After removing 743 subjects (125 died, 618 moved away, five had severe medical problems), 6265 inhabitants were invited. Of those, 4308 subjects participated in the baseline study (response 68.8%). The five-year-follow-up (SHIP-1) included 3300 subjects with examinations held between 2002 and 2006. Out of this collective, subjects for this study were selected, as detailed below. The SHIP study protocol was approved by the local Ethics committee of the University of Greifswald and all participants gave informed written consent.

### Covariates

Sociodemographic, behavioral and medical variables were assessed by computer-assisted personal interviews. School education level was categorized based on the East German three-level school system (<10, 10, >10 years). Smoking status was defined as never, former and current smoking. Diabetes mellitus was defined as self-reported physician's diagnosis or intake of anti-diabetic medication (Anatomical Therapeutic Chemical (ATC) A10). Body height and weight were determined using calibrated scales, and body mass index (BMI) was calculated by kilogram divided by square meter. The BMI was categorized as <25, ≥25 but <30 and ≥30 kg/m^2^.

### Periodontal examination

Periodontal examinations comprised clinical attachment loss (CAL) and probing depth (PD). Measurements were assessed at four surfaces per tooth (distobuccal, midbuccal, mesiobuccal and midlingual/midpalatinal) according to the half-mouth method, alternating on the left or right side. Third molars were excluded. A periodontal probe was used (PCP-2, Hu-Friedy, Chicago, IL, USA). Mean CAL and mean PD were calculated.

Biannual calibration exercises were conducted on subjects not associated with the study yielding intra-class correlations between 0.70–0.89 per examiner and an inter-rater correlation of 0.90 for CAL measurements.

### Selection of study subjects

Inclusion criteria for this study were: 35–54 years of age; no periodontal treatment within the last five years; complete dental status; >10 natural teeth; complete information on smoking status, education, diabetes and BMI. Subjects were defined as periodontally diseased, if their mean CAL and mean PD ranged within the highest quartile calculated separately within sex and 5-year-age-categories. Accordingly, subjects were defined as periodontally healthy, if their mean CAL and mean PD ranged within the lowest quartile calculated separately within sex and 5-year-age-categories. One hundred periodontally diseased subjects (referred henceforth as “cases”) were randomly selected such that the number of cases was uniformly distributed across possible combinations of age (5-year age groups) and gender, resulting in 12–14 cases per subgroup. Applying a 1∶1 matching according to age (5-year age groups) and gender, 100 periodontally healthy subjects (referred henceforth as “controls”) were randomly selected. Twelve pairs were excluded because samples of at least one of the paired subjects were either not available or DNA could not be recovered due to technical reasons. Finally, tongue scrapings of 88 matched pairs were analyzed.

### Sample collection and DNA isolation

Tongue biofilm was taken from the middle third of the tongue dorsum with a sterile spatula. The spatula was transferred into 2.0 ml of phosphate-buffered saline (PBS) and after shaking vigorously for 30 s the spatula was removed. Microbial suspensions in PBS were kept at −80°C until further processed. Prior to DNA isolation 1.2 ml of each suspension was centrifuged at 10,000xg for 15 min. Then 1.1 ml of the supernatant was discarded and the pellet was resuspended in the remaining 100 µl. After adding 130 µl of lysis buffer and 20 µl of proteinase K (both MagNA Pure LC DNA Isolation Kit III, Roche, Mannheim, Germany) the mixture was incubated at 65°C for 10 min followed by 95°C for 10 min.

After the pre-isolation steps as described above, DNA was extracted and purified using the automated MagNA Pure LC platform (Roche, Mannheim, Germany) based on binding of nucleic acids on the surface of magnetic glass particles provided by the MagNA Pure LC DNA Isolation Kit III (Roche, Mannheim, Germany). The standard isolation protocol according to the manufacturers' instructions was applied with a sample volume of 250 µl and a final elution volume of 150 µl. DNA was extracted from September 2010 until February 2011 and samples were stored at −20°C until usage.

### Oligonucleotide selection and design

The species-specific primers and probes applied in the qPCR assay targeted the following single copy genes: arg-gingipain of *P. gingivalis* [28]; leukotoxin C of *A. actinomycetemcomitans* [28]; β-subunit of RNA polymerase of *F. nucleatum*, and glycosyl transferase P of *S. sanguinis* [29]. The domain-specific oligonucleotides were based on conserved regions of bacterial [30] and archaeal [31] 16S rRNA genes. Previously published primer and probe sequences were reevaluated by comparing entries accessible in the nucleotide database of the National Center for Biotechnology Information (NCBI, http://www.ncbi.nlm.nih.gov) by using the computer algorithm Basic Local Alignment Search Tool (BLAST) [32].

The oligonucleotide set specific for *F. nucleatum* and the probe for *S. sanguinis* were designed using the computer program Primer3 [33] and were, together with three modified oligonucleotides highlighted in Table 1, subsequently validated for uniqueness by performing a NCBI BLAST search and for absence of any hairpin formation, complementary and self-annealing by applying the software OligoCalc [34] and mfold [35]. Novel primers were checked empirically for PCR artefact formation such as primer dimers by melting curve analysis using the SYBR Green 1 Master kit (Roche Applied Science, Mannheim, Germany). Moreover, specificity of all primer sets were validated in endpoint PCR experiments run in parallel using genomic DNA from *P. gingivalis* DSM 20709, *A. actinomycetemcomitans* DSM 8324, *F. nucleatum* DSM 15643, *S. sanguinis* DSM 20567, *Methanobrevibacter oralis* DSM 7256 and *Treponema denticola* DSM 14222. Additionally, *F. naviforme* DSM 20699 and *F. necrophorum* DSM 20698 which are phylogenetically closely related to *F. nucleatum* [36] were used to validate the primer set specifically designed for *F. nucleatum*.

**Table 1:** Sequence information, specificity, amplicon size and molarity of oligonucleotide primers and probes used in the qPCR assays.

Taxon	Oligonucleotide sequence (5' ->3') ^a^	Target gene and amplicon size	Reference	Molarity
*Porphyromonas gingivalis*	F: AGGATCGCTCAGCGTAGCATT ^c^	Arg-gingipain	[28]	0.10 µM
	R: CCTACGTGTACGGACAGAGCTATA ^c^	(Rgp), 71 bp	[28]	0.50 µM
	P: FAM-TCGCCCGGGAAGAACTTGTCTTCA-BHQ1		[28]	0.10 µM
*Aggregatibacter actinomycetemcomitans*	F: ACGCAGACGATTAACTGAATTTAA ^b^	Leukotoxin C	This study	0.10 µM
	R: GATCTTCACAGCTATATGGCAGCTA	(*lktC*), 77 bp	[28]	0.25 µM
	P: FAM-TTACCCTTCTACCGTTGCCATGGG-BHQ1 ^b^		This study	0.10 µM
*Fusobacterium nucleatum*	F: TGGCATAGCTTCACCTTTGA	β-subunit of RNA polymerase	This study	0.25 µM
	R: CAAAGACTTGGGGAAATGGA	(*rpoB*), 144 bp	This study	0.50 µM
	P: FAM-TGCTCCATAAGCTTCCAATGCCCA-BHQ1		This study	0.10 µM
*Streptococcus sanguinis*	F: GGCGCCTGTTAATACTGAGC	Glycosyl transferase P	[29]	0.10 µM
	R: GTTTTTCCATCCTTGAGGATAGC	(*gtpP*), 330 bp	[29]	0.50 µM
	P: FAM-TCGATGCAGAGACCGGAGCC-BHQ1		This study	0.10 µM
*Archaea*	F: CGGTGAATAYGYCCCTGC	16S ribosomal RNA	[31]	0.25 µM
	R: AAGGAGGTGATCCRGCCGCA	(16S rRNA), 173 bp	[31]	1.00 µM
	P: FAM-CTTGCACACACCGCCCGTC-BHQ1 ^b^		This study	0.10 µM
*Bacteria*	F: TGGAGCATGTGGTTTAATTCGA	16S ribosomal RNA	[30]	0.25 µM
	R: TGCGGGACTTAACCCAACA	(16S rRNA), 159 bp	[30]	0.10 µM
	P: FAM-CACGAGCTGACGACARCCATGCA-BHQ1		[30]	0.10 µM

a) F, forward primer; R, reverse primer; P, probe; FAM, 6-carboxyfluorescein; BHQ1, Black Hole Quencher 1.

b) Published oligonucleotide sequences showing mismatches were modified by using the nucleotides which are underlined at these positions.

c) In their study, Morillo and colleagues [28] designated the forward primer as reverse and the other way around. Here, the correct designation is given.

Primer titration were performed in symmetric and asymmetric qPCR providing the same and different ratio of forward and reverse primers at final concentrations of 50, 100, 250, 500, and 1000 nM. The qPCR assay was as depicted below except that no BSA was added, 200 nM of probe and 1•10^5^ plasmid copies were used. Successive probe titrations were conducted with the optimized forward and reverse primer ratio at final concentrations of 50, 100, 150 and 200 nM.

Oligonucleotides were synthesized by MWG-Biotech (Ebersberg, Germany). Probes were labeled with 6-carboxyfluorescein (FAM) at its 5′ end and Black Hole Quencher 1 (BHQ1) at its 3′ position.

### Plasmid standards

Target sequences defined by the described primers (Table 1) were generated and cloned into the pSC-B-amp/kan vector using the Strata Clone Ultra Blunt PCR Cloning Kit (Stratagene, La Jolla, USA), which couples PCR amplification with topoisomerase-based PCR cloning. The following strains were used: *S. sanguinis* DSM 20567 (target genes were bacterial 16S ribosomal RNA and glycosyl transferase P), *M. oralis* DSM 7256 (archaeal 16S ribosomal RNA), *F. nucleatum* DSM 15643 (β-subunit of RNA polymerase), *P. gingivalis* DSM 20709 (arg-gingipain), and *A. actinomycetemcomitans* DSM 8324 (leukotoxin C). Preparations were carried out according to the manufacturer standard protocol for targets below 10 kb and the plasmids were then transformed into heat shock competent cells provided by the same kit.

Each plasmid was purified from 3 ml of an overnight cell culture with the Charge Switch Plasmid ER Mini Kit (Invitrogen, Carlsbad, USA) and sequenced by MWG-Eurofins (Ebersberg, Germany). The plasmid concentrations were quantified using a micro spectrophotometer (NanoDrop 1000; Thermo Scientific, Wilmington, USA).

### Endpoint PCR assay

Initially, primer were tested in a 25 µl PCR mixtures consisting of 1× Fast Start High Fidelity, 2 mM MgCl_2_, 200 µM dNTPs, 1.25 units of Fast Start High Fidelity enzyme blend (all Roche, Mannheim, Germany); 2.0% (w/v) of dimethyl sulfoxide (DMSO); 500 nM of each primer; and genomic DNA as template. The following PCR conditions were applied: an initial denaturation step at 95°C for 120 s; 35 cycles denaturation at 95°C for 30 s, annealing at 60°C for 30 s, and extension at 72°C for 30 s; followed by a final extension step for 300 s.

Amplified DNA fragments were subjected to electrophoresis in ethidium bromide 2% (v/w) agarose gels and were visually inspected for presence of appropriate-sized PCR products.

### Quantitative PCR assay

A single qPCR reaction mixture of 20 µl final volume consisted of 1× Light Cycler 480 Probes Master (Roche, Mannheim, Germany) or 1× Maxima Probe qPCR Master Mix (Fermentas, St. Leon-Rot, Germany); forward and reverse primers at concentration as listed in Table 1 and 100 nM of the corresponding probe (MWG-Biotech, Ebersberg, Germany); 8 µg of non-acetylated bovine serum albumin (BSA; Sigma-Aldrich, St. Louis, USA); and 5 µl of DNA or plasmid as template. All samples were analyzed in triplicates. Amplification and detection were conducted in plates in 96 well formats using a Light Cycler 480 instrument II (Roche, Mannheim, Germany). Thermal conditions started with 95°C for 10 min to activate the Fast Start *Taq* DNA polymerase, followed by 50 cycles with denaturation at 95°C for 15 s and amplification at 60°C for 30 s. The cycle threshold (C*_t_*), defined as the number of amplification cycle at which the signal intensity crosses background fluorescence, was calculated by applying the ‘Second Derivative Maximum’ algorithm (Light Cycler 480 Software release 1.5.0) which identifies the maximum of the second derivative of the amplification curve. Background fluorescence was automatically corrected by the software by subtracting the mean fluorescence of the qPCR cycles 2 to 6 from the fluorescence values. The sensitivity and efficiency of each of the six qPCR assays were determined by using serial diluted plasmid DNA as templates to generate primarily more than six individual standard curves for each qPCR assay (Figure S1) and standard curves in each individual qPCR run. Efficient qPCR amplification in all qPCR-runs was ensured by efficiencies between 1.9 and 2.1.

### Statistical analyses

To determine the abundance for *P. gingivalis*, *F. nucleatum*, *A. actinomycetemcomitans* and *S. sanguinis*, we divided the single species count by the detected number of 16S-rRNA gene copies per sample (referred henceforth as ‘abundance’). Since the estimation-models for species count within a sample are still controversial [38], [39], we decided to use the 16S-rRNA gene copies as denominator without mathematical correction well aware that this could lead to an overestimation of *Bacteria* and *Archaea* inside the sample. The bacterial as well as the archaeal abundance was calculated by dividing the respective 16S rRNA gene count by ng extracted DNA. The portion of *Archaea* within the total prokaryotes was calculated with the following formula: % *Archaea*  =  (archaeal 16S rRNA gene count/(archaeal 16S rRNA gene count + archaeal 16S rRNA gene count)) * 100.

Since abundances were not normally distributed, medians with interquartile ranges (IQR; 25%-75% quantile) were presented. Wilcoxon matched-pairs signed-ranks test or exact McNemar-tests were used to determine differences in variable distributions between cases and controls. Crude Odds Ratios (ORs) with exact 95% confidence intervals (CI) were determined.

Conditional logistic regression was used to evaluate the association between bacterial detection (yes/no) or abundances (exposures) and periodontal status (dependent variable; cases versus controls). Abundance values were categorized into tertiles (T1–T3). These tertiles were calculated using the following 33.33% and 66.67% quantiles: 0 and 9.28*10^−3^ for *P. gingivalis* abundances, 0 and 1.1*10^−4^ for *A. actinomycetemcomitans* abundances, 0 and 2.162*10^−2^ for *F. nucleatum* abundances, 0 and 2.77*10^−3^ for *S. sanguinis* abundances, 0 and 5.5*10^2^ for archaea abundances and 1.8*10^6^ and 2.6*10^6^ for bacterial abundances. ORs with their 95% CIs were calculated. All models were adjusted for age (continuously), gender, school education, smoking status and BMI.

Data analyses were performed using STATA/SE 12.0 [37]. P values <0.05 were considered statistically significant.

## Results

### Specificity and sensitivity of qPCR assays

*In silico* analysis of the published oligonucleotide sequences revealed mismatches and resulted in modified oligonucleotides for *A. actinomycetemcomitans* [28] and *Archaea* [31]. Modifications are indicated in Table 1. Primer sets were evaluated empirically in PCR experiments applying DNA extracted from *S. sanguinis* DSM 20567, *F. nucleatum* DSM 15643, *P. gingivalis* DSM 20709, *A. actinomycetemcomitans* DSM 8324, *M. oralis* DSM 7256, *T. denticola* DSM 14222, *F. naviforme* DSM 20699 and *F. necrophorum* DSM 20698. Neither false-negative results nor cross-reactivity among the tested species were observed (data not shown). All designed and modified oligonucleotides showed no secondary structures when analyzed by the software OligoCalc and mfold and melting curve analysis demonstrated further the absence of any PCR artifact formation for each of the primer sets (data not shown).

For each qPCR assay standard curves showed a strong linear inverse relationship (*R^2^*>99%) between the cycle threshold values and the log_10_ target gene numbers over several orders of magnitude (Figure S1). Analytical sensitivity for the species-specific amplification reactions were 100 cells per PCR for *P. gingivalis*, and 10 cells for *F. nucleatum*, *A. actinomycetemcomitans*, and *S. sanguinis*, respectively. The amplification of the qPCR assays displayed efficiency values within the recommended range of 90% to 110%.

### Characteristics of study subjects

We analyzed the data of 88 matched pairs. Cases and controls differed significantly regarding school education, smoking status and BMI (p≤0.01, Table 2). Cases had higher values of mean PD and mean CAL and fewer teeth compared with controls (p<0.001).

**Table 2:** Subject characteristics.

	Controls	Cases	p value 1
N	88	88	
Age, years	45.1±5.9	45.1±5.6	0.74
Male gender	44 (50.0%)	44 (50.0%)	-
School education			
<10 years	8 (9.1%)	19 (21.6%)	
10 years	52 (59.1%)	60 (68.2%)	
>10 years	28 (31.8%)	9 (10.2%)	<0.001
Smoking status			
Never	38 (43.2%)	20 (22.7%)	
Former	27 (30.7%)	17 (19.3%)	
Current	23 (26.1%)	51 (57.0%)	<0.001
Diabetes (yes)	3 (3.4%)	5 (5.7%)	0.73
BMI, kg/m^2^	26.7±5.2	28.1±5.3	0.01
Mean PD, mm	1.69±0.14	3.24±0.63	<0.001
Mean CAL, mm	0.43±0.34	3.71±1.20	<0.001
Tooth count	24.4±3.0	20.9±4.7	<0.001

Data are presented as numbers (percentages) or mean ± SD.

1 paired t-test or McNemar test.

CAL, clinical attachment loss; PD, probing depth.

### Qualitative detection of oral microorganisms in tongue scrapings from cases and controls

The overall number of bacterial species identified per subject differed significantly between cases and controls (p = 0.03). Three or four bacterial species were more often identified in cases (44.3%) than in controls (28.4%, p = 0.03). None of the bacterial species was detected in 1.1% of the cases and in 9.9% of the controls.

Associations between the detection of bacterial species and periodontal disease status are shown in Table 3. *P. gingivalis* was significantly more often detected in cases (OR 3.7; 95%-CI 1.8–8.3; p<0.001) compared to controls. For *F. nucleatum* and archaeal sequences borderline significances with ORs of 1.7 (95%-CI 0.9–3.4; p = 0.13) and 0.6 (95%-CI 0.3–1.1; p = 0.14), respectively, were observed.

**Table 3:** Crude associations between periodontal status (periodontally healthy (controls) versus periodontally diseased subjects (cases)) and detection of different pathogens (yes/no) in tongue scrapings.

	Cases	Controls			
*P. gingivalis*		Detection	No detection	Sum	
	Detection	27 (30.7%)	37 (42.0%)	64 (72.7%)	p<0.001^1^
	No detection	10 (11.4%)	14 (15.9%)	24 (27.3%)	OR = 3.7 (1.8; 8.3)
	Sum	37 (42.0%)	51 (58.0%)	88 (100%)	
*A. actinomycetemcomitans*					
	Detection	20 (22.7%)	18 (20.5%)	38 (43.2%)	p = 0.29 1
	No detection	26 (29.5%)	24 (27.3%)	50 (56.8%)	OR = 0.7 (0.4; 1.3)
	Sum	46 (52.3%)	42 (47.7%)	88 (100%)	
*F. nucleatum*					
	Detection	20 (22.7%)	27 (30.7%)	47 (53.4%)	p = 0.13 1
	No detection	16 (18.2%)	25 (28.4%)	41 (46.6%)	OR = 1.7 (0.9; 3.4)
	Sum	36 (40.9%)	52 (59.1%)	88 (100%)	
*S. sanguinis*					
	Detection	27 (30.7%)	22 (25.0%)	49 (55.7%)	p = 0.77 1
	No detection	25 (28.4%)	14 (15.9%)	39 (44.3%)	OR = 0.9 (0.5; 1.6)
	Sum	52 (59.1%)	36 (40.9%)	88 (100%)	
Archaea					
	Detection	23 (26.1%)	17 (19.3%)	40 (45.5%)	p = 0.14^1^
	No detection	28 (31.8%)	20 (22.7%)	48 (54.5%)	OR = 0.6 (0.3; 1.1)
	Sum	51 (58.0%)	37 (42.0%)	88 (100%)	
Bacteria					
	Detection	88 (100%)	0 (0%)	88 (100%)	NA
	No detection	0 (0%)	0 (0%)	0 (0%)	NA
	Sum	88 (100%)	0 (0%)	88 (100%)	

1 Exact McNemar Test.

NA, not annotated; OR, Odds Ratio.

Higher detection rates of *P. gingivalis* in cases were confirmed in all subgroups defined by age and gender (Tables S1 and S2 in File S1). For certain subgroups, significant or borderline significant associations between detection of *A. actinomycetemcomitans*, *F. nucleatum* or *Archaea* and periodontal status are displayed in Tables S1 and S2 in File S1.

### Quantification of oral microorganisms in tongue scrapings from cases and controls

Abundance of *P. gingivalis* (p<0.001) and the overall bacterial abundance (p = 0.02) were significantly higher in cases compared with controls (Table 4). Abundances for remaining species did not differ significantly between cases and controls.

**Table 4:** Comparison of prokaryotic abundances between periodontally healthy (controls) and periodontally diseased subjects (cases). N = 88 pairs.

	Controls	Cases	P-value 1
*P. gingivalis*	0 (0; 2.3⋅10^−5^)	1.1⋅10^−4^ (0; 6.6⋅10^−4^)	<0.001
*A. actinomycetemcomitans*	8.3⋅10^−8^ (0; 2.8⋅10^−6^)	0 (0; 3.9⋅10^−6^)	0.53
*F. nucleatum*	0 (0; 7.0⋅10^−4^)	2.3⋅10^−5^ (0; 6.8⋅10^−4^)	0.33
*S. sanguinis*	4.5⋅10^−6^ (0; 5.2⋅10^−5^)	2.3⋅10^−6^ (0; 3.6⋅10^−5^)	0.29
Archaea^2^	0.2⋅10^3^ (0; 0.6⋅10^3^)	0 (0; 1.6⋅10^3^)	0.48
% Archaea^3^	0 (0; 4.4⋅10^−2^)	9.0⋅10^−3^ (0; 2.5⋅10^−2^)	0.52
Bacteria 1	2.1⋅10^6^ (1.5⋅10^6^; 2.7⋅10^6^)	2.5⋅10^6^ (1.7⋅10^6^; 3.6⋅10^6^)	0.02

Data are presented as median (25%; 75% quantile).

1 Wilcoxon matched-pairs signed-ranks test.

2 proportion of 16S rRNA gene copies per ng extracted DNA; N, number of matched pairs.

3 percent of archaeal 16S rRNA gene copies per prokaryotic 16S rRNA gene copies (Archaea+Bacteria)*100.

Age and gender dependent stratification of the study population revealed higher abundances of *P. gingivalis* in cases compared to controls within all subgroups (Table S3 in File S1). Abundances of *A. actinomycetemcomitans*, *F. nucleatum*, *S. sanguinis* differed significantly between cases and controls within selected subgroups only.

### Association between detection of bacterial species and periodontal status

In fully adjusted models (Table 5), *P. gingivalis* detection was strongly associated with periodontal disease (OR 3.60 (95%-CI 1.47–8.86), p = 0.005). Also, *A. actinomycetemcomitans* detection was significantly associated with periodontal health (OR 0.41 (95%-CI 0.18–0.97), p = 0.04). No significant associations were found for the other bacteria or archaea. Results for *P. gingivalis* were confirmed in most gender and age stratified subgroups (Table S4 in File S1). Within females, significant associations of *A. actinomycetemcomitans* with periodontal health and of *F. nucleatum* with periodontal disease were found (Table S4 in File S1).

**Table 5:** Adjusted Odds Ratios quantifying chance of being periodontally diseased depending on detection (yes/no) or abundance of different pathogens in tongue scrapings in the overall study population (N = 88 pairs).

	Detection (yes/no)		Abundance ◊			
			OR (95% CI)			
Group/Species	OR (95% CI)	P value	T1	T2	T3	P for trend
*P. gingivalis*	3.60 (1.47; 8.86)	0.005	1.00	0.99 (0.34; 2.93)	17.45 (4.00; 76.04) ***	<0.001
*A. actinomycetemcomitans*	0.41 (0.18; 0.97)	0.04	1.00	0.13 (0.03; 0.59) **	0.61 (0.24; 1.54)	0.19
*F. nucleatum*	1.22 (0.54; 2.74)	0.64	1.00	1.55 (0.39; 6.22)	1.15 (0.49; 2.70)	0.75
*S. sanguinis*	0.71 (0.34; 1.47)	0.35	1.00	1.19 (0.45; 3.10)	0.49 (0.20; 1.17)	0.13
Archaea^1^	0.77 (0.36; 1.62)	0.49	1.00	0.10 (0.02; 0.47) **	2.02 (0.75; 5.46)	0.26
% Archaea^2^	-	-	1.00	0.22 (0.06; 0.74) *	1.25 (0.53; 2.95)	0.58
Bacteria^1^	NA	NA	1.00	1.21 (0.45; 3.25)	2.64 (0.99; 7.08)	0.048

Conditional logistic regression modeling periodontal status (cases versus controls, dependent variable) on detection (yes/no) or abundances adjusting for age (cont.), school education, smoking status and BMI.

◊ Abundances were categorized as tertiles (T1–T3). Numbers within tertiles were: *P. gingivalis*: 75-43-58, *A. actinomycetemcomitans*: 92-26-58, *F. nucleatum*: 93-25-58, *S. sanguinis*: 75-43-58, Archaea 85-33-58, %Archaea: 85-33-58, Bacteria: 59-59-58.

1 proportion of 16S rRNA gene copies per ng extracted DNA; N, number of matched pairs.

2 percent of archaeal 16S rRNA gene copies per prokaryotic 16S rRNA gene copies (Archaea+Bacteria)*100.

*p<0.05, ** p<0.01, *** p<0.001 versus reference group.

### Association between bacterial abundance and periodontal status

In fully adjusted models, the risk of periodontal disease raised significantly with elevated *P. gingivalis* abundances (p for trend<0.001, Table 5). A very high abundance of *P. gingivalis* (T3) was associated with a 17-fold increased risk for periodontal disease compared with the reference group (OR 17.45 (95%-CI 4.00–76.04, p<0.001). Moderate abundances of *A. actinomycetemcomitans* (T2) (OR 0.13 (95%-CI 0.03–0.59, p<0.01)) were significantly associated with reduced risk for periodontal disease compared to subjects with low abundances. For high abundances (T3) this protective effect slightly leveled off. For archaeal abundances associations were U-shaped. Moderate archaeal abundances were significantly health associated compared to subjects with low abundances (OR 0.10 (95%-CI 0.02–0.47, p<0.01)), while high abundances (T3) showed a tendency towards higher, but non-significant, ORs indicating a putatively disease associated effect. Considering only the proportion of *Archaea* within all prokaryotes revealed the same results. This was not detectable using un-normalized data (Table S5 in File S1). Furthermore, risk for periodontal disease increased significantly across categories for bacterial abundance (p for trend = 0.048). However, differences between groups were non-significant (Table 5).

## Discussion

The comparison of oral microbiological data across various studies is often difficult because the tested oral habitats, sampling procedures and microbiological methods differ (24, 25, 28, 47, 48, 52–54). If molecular methods are applied for microbial detection, results depend on the DNA extraction method applied, the selection of microbial targets, and if normalization of quantitative data is performed to correct for sample errors [38]–[40]. In this study, we used single copy genes for microbial species identification and applied an automated nucleic acid extraction protocol. Furthermore, we normalized quantitative microbial DNA data using either total bacterial DNA or total DNA from single samples. In agreement with a study by Matarazzo et al. we also detected higher bacterial proportion in periodontally diseased subjects [3]. We found that the risk of periodontal disease significantly increased for higher total bacterial abundance.

In our study, overall detection rates of *P. gingivalis* and *A. actinomycetemcomitans* from tongue scrapings were 57.4% and 47.7%, respectively. In agreement with previous studies we found a significantly higher detection rate of *P. gingivalis* in periodontally diseased subjects [41]. Comparable detection rates for both species in saliva were reported in a Finnish study with 84 periodontally diseased subjects [41]. We found no significant difference in the detection rates of *A. actinomycetemcomitans* on the tongue in contrast to previous studies sampling different oral habitats [24], [41].

Results for abundance data for *P. gingivalis* are in agreement with un-normalized data from saliva and tongue scrapings [21], [42] showing an increased amount of *P. gingivalis* in diseased subjects. Indeed, the median abundance of *P. gingivalis* in *P. gingivalis*-positive and diseased subjects found in this study was comparable to data published by Kubinowa *et al.* [8]. Additionally, we could demonstrate that the risk of periodontal disease is significantly increased with higher *P. gingivalis* abundance.

Interestingly, we detected a health-associated correlation of low *A. actinomycetemcomitans* abundances on the tongue. Kubinowa and colleagues did not categorize their abundance data and detected no significant correlation between *A. actinomycetemcomitans* abundance and periodontal status on the tongue [8]. Aside from different cohort sizes, this discrepancy might also be explained by an uneven distribution of *A. actinomycetemcomitans* strains and serotypes in the population and their impact on periodontal disease and occurrence of other bacteria [42]–[45].

In our study detection rates of *S. sanguinis* (55.7% in cases and 59.1% in controls) did not differ significantly between cases and control. Interestingly, detection rates were higher compared to a previous study [46]. In line with this, neither the detection rates nor abundances were significantly different between cases and controls in previous studies sampling different oral habitats [47], [48]. In agreement with a previous study there was no significant difference in colonization of *F. nucleatum* on the tongue between healthy and diseased subjects [47], [48]. Although, differences between healthy and diseased subjects were recorded in subgingival plaques [49].

In previous studies members of the archaeal domain were detected in subgingival plaque and on teeth ([50] and references within), while the diversity seemed to be very small [3]. In contrast to the study of Lepp and colleagues [2], we detected archaeal sequences in tongue scrapings of periodontally healthy and diseased subjects. The observed detection rates of archaeal sequences in tongue scrapings (45.5% of cases and 58.0% of controls) were within the range of detection rates reported for subgingival samples ranging from 36% [2] to 96.4% [3]. In agreement with the results for detection rates of *Archaea* in subgingival samples [3], detection rates on tongue scrapings did not differ significantly between periodontally healthy and diseased subjects in our study. However, our data showed that moderate *Archaea* abundances were associated with a healthy periodontal status on the tongue. High abundances suggested disease-related effects. Consistently, previous studies reported increased subgingival levels of *Archaea* or methanogenic *Archaea* in generalized aggressive periodontitis or aggressive periodontitis, respectively [3], [51].

These observations indicate a Janus-faced role of *Archaea* in oral health. Colonization of the tongue at low or moderate levels is probably related to a healthy periodontal status whereas high abundances correlate with periodontal disease. A difference in archaeal species or phylotype colonization, species abundance and different oral habitats may also provide a possible explanation for this phenomenon. Because of time- and cost intensiveness of full-mouth recordings [52], periodontal measurements within SHIP were taken according to the half-mouth method at four sites per tooth. Based on the same premise, tongue scrapings were collected in SHIP-1. Taking tongue samples is reasonable; because the tongue might serve as a global reservoir for oral microorganisms and periodontitis associated microbes can frequently be detected in this habitat [2], [25], [47], [53]–[55]. Microbes inhabiting the dorsal tip of the tongue could disseminate into the oral cavity, which might lead to recolonization of the gingival sulcus resulting in periodontal disease. However, periodontal microbes might be less frequently detected in tongue samples than in subgingival samples [47], [56], which is considered to be the primary niche of periodontal pathogens [47]. This might have led to an underestimation of periodontal disease severity [57] and might have shifted effect estimates towards the null association [58]. When evaluating associations between bacterial abundances and periodontal status in matched pairs, analyses had to be adjusted for periodontal risk factors, including age, school education, smoking status and BMI, to avoid confounding. Because gender was evenly distributed in controls and cases, we did not adjust for it. Overall, we tried to avoid over-adjustment to reduce bias related to an increased number of variables, having in mind that sample size was limited in this study [59]. Further, the number of events per variable (>10) and the number of discordant matched sets (N≥10) were sufficient in overall analyses. Still these aspects contributed to large confidence intervals in multivariate models. Nevertheless, key associations remained after adjustment for confounders in multivariate models. Both aspects contributed to large confidence intervals in multivariate models. However, our study illustrates that a categorization of microbial abundances unravels associations with periodontal status not seen with detection rate analyses or un-normalized data alone.

## Supporting Information

Figure S1 **Standard curves with standard deviation of qPCR assay based on serial dilution of plasmid DNA.** The equations and the square regression coefficient (*R^2^*) of the standard curve and the efficiency of amplification reactions are given.
(TIF)

File S1 **This file contains Tables S1–S5.**
(DOC)
