# Supplementary material for: Quantitative Molecular Detection of Putative Periodontal Pathogens in Clinically Healthy and Periodontally Diseased Subjects
Source: PLoS One. 2014 Jul 16;9(7):e99244. doi: 10.1371/journal.pone.0099244 (PMC4100758; doi:10.1371/journal.pone.0099244)
Supplement: File S1 — This file contains Tables S1–S5. (DOC) [file pone.0099244.s002.doc]

**Supporting Table S1.** Association between periodontal status (periodontally healthy (controls) versus periodontally diseased subjects (cases)) and detection of different pathogens (yes/no) in tongue scrapings in 35-44- and 45-54-aged subjects.

| **Group** | **Taxon** | **Cases** | **Controls** | |  |  |
| --- | --- | --- | --- | --- | --- | --- |
|  |  |  | **Detection** | **No detection** | **Sum** |  |
| 35-44 years (N=43) | *P. gingivalis* | Detection | 10 (23.3%) | 17 (39.5%) | 27 (62.8%) | **p=0.007 ^1^** |
|  |  | No detection | 4 (9.3%) | 12 (27.9%) | 16 (37.2%) | **OR=4.3** |
|  |  | Sum | 14 (32.6%) | 29 (65.9%) | 43 (100%) | **(1.4; 17.4)** |
|  |  |  |  |  |  |  |
|  | *A. actinomycetemcomitans* | Detection | 8 (18.6%) | 7 (16.3%) | 15 (34.9%) | p=0.19 **^1^** |
|  |  | No detection | 14 (32.6%) | 14 (32.6%) | 28 (65.1%) | OR=0.5 |
|  |  | Sum | 22 (51.2%) | 21 (48.8%) | 43 (100%) | (0.2; 1.3) |
|  |  |  |  |  |  |  |
|  | *F. nucleatum* | Detection | 9 (20.5%) | 13 (30.2%) | 22 (51.2%) | p=0.52**^1^** |
|  |  | No detection | 9 (20.5%) | 12 (27.9%) | 21 (48.8%) | OR=1.4 |
|  |  | Sum | 18 (41.9%) | 25 (58.1%) | 43 (100%) | (0.6; 3.8) |
|  |  |  |  |  |  |  |
|  | *S. sanguinis* | Detection | 13 (30.2%) | 11 (25.6%) | 24 (55.8%) | p=1.00**^1^** |
|  |  | No detection | 11 (25.6%) | 8 (18.2%) | 19 (44.2%) | OR=1.0 |
|  |  | Sum | 24 (55.8%) | 19 (44.2%) | 43 (100%) | (0.4; 2.5) |
|  |  |  |  |  |  |  |
|  | Archaea | Detection | 8 (18.6%) | 7 (16.3%) | 15 (34.9%) | p=0.09 **^1^** |
|  |  | No detection | 16 (37.2%) | 12 (27.9%) | 28 (65.1%) | OR=0.4 |
|  |  | Sum | 24 (55.8%) | 19 (44.2%) | 43 (100%) | (0.2; 1.1) |
|  |  |  |  | |  |  |
|  |  |  | **Detection** | **No detection** | **Sum** |  |
| 45-54 years (N=45) | *P. gingivalis* | Detection | 17 (37.8%) | 20 (44.4%) | 37 (82.2%) | **p=0.009 ^1^** |
|  |  | No detection | 6 (13.3%) | 2 (4.5%) | 8 (17.8%) | **OR=3.3** |
|  |  | Sum | 23 (51.1%) | 22 (48.9%) | 45 (100%) | **(1.3; 10.1)** |
|  |  |  |  |  |  |  |
|  | *A. actinomycetemcomitans* | Detection | 12 (26.7%) | 11 (24.4%) | 23 (51.1%) | p=1.00 **^1^** |
|  |  | No detection | 12 (26.7%) | 10 (22.2%) | 22 (48.9%) | OR=0.9 |
|  |  | Sum | 24 (53.3%) | 21 (46.7%) | 45 (100%) | (0.4; 2.3) |
|  |  |  |  |  |  |  |
|  | *F. nucleatum* | Detection | 11 (24.4%) | 14 (31.1%) | 25 (55.6%) | p=0.19 **^1^** |
|  |  | No detection | 7 (15.6%) | 13 (28.9%) | 20 (44.4%) | OR=2.0 |
|  |  | Sum | 18 (40.0%) | 27 (60.0%) | 45 (100%) | (0.8; 5.9) |
|  |  |  |  |  |  |  |
|  | *S. sanguinis* | Detection | 14 (31.1%) | 11 (24.5%) | 25 (55.6%) | p=0.69**^1^** |
|  |  | No detection | 14 (31.1%) | 6 (13.3%) | 20 (44.4%) | OR=0.8 |
|  |  | Sum | 28 (62.2%) | 17 (37.8%) | 45 (100%) | (0.3; 1.9) |
|  |  |  |  |  |  |  |
|  | Archaea | Detection | 15 (33.3%) | 10 (22.2%) | 25 (55.5%) | p=0.83 **^1^** |
|  |  | no detection | 12 (26.7%) | 8 (17.8%) | 20 (44.5%) | OR=0.8 |
|  |  | sum | 27 (60.0%) | 18 (40.0%) | 45 (100%) | (0.3; 2.1) |

**^1^** Exact McNemar Test; 95% confidence interval (CI) for Odds Ratio (OR) is exact.

N, Number of matched pairs.

**Supporting Table S2.** Association between periodontal status (periodontally healthy (controls) versus periodontally diseased subjects (cases)) and detection of different pathogens (yes/no) in tongue scrapings in females and males.

| **Group** | **Taxon** | **Cases** | **Controls** | |  |  |
| --- | --- | --- | --- | --- | --- | --- |
|  |  |  | **Detection** | **No detection** | **Sum** |  |
| Females (N=44) | *P. gingivalis* | Detection | 14 (31.8%) | 17 (38.6%) | 31 (70.5%) | **p=0.02 ^1^** |
|  |  | No detection | 5 (11.4%) | 8 (18.2%) | 13 (29.5%) | **OR=3.4** |
|  |  | Sum | 19 (43.2%) | 25 (56.8%) | 44 (100%) | **(1.2; 11.8)** |
|  |  |  |  |  |  |  |
|  | *A. actinomycetemcomitans* | Detection | 11 (25.0%) | 5 (11.4%) | 16 (36.4%) | **p=0.007 ^1^** |
|  |  | No detection | 19 (43.2%) | 9 (20.4%) | 28 (63.6%) | **OR=0.3** |
|  |  | Sum | 30 (68.2%) | 14 (31.8%) | 44 (100%) | **(0.1; 0.7)** |
|  |  |  |  |  |  |  |
|  | *F. nucleatum* | Detection | 13 (29.5%) | 14 (31.8%) | 27 (61.4%) | p=0.06 **^1^** |
|  |  | No detection | 5 (11.4%) | 12 (27.3%) | 17 (38.6%) | OR=2.8 |
|  |  | Sum | 18 (40.9%) | 26 (59.1%) | 44 (100%) | (0.95; 9.9) |
|  |  |  |  |  |  |  |
|  | *S. sanguinis* | Detection | 18 (40.9%) | 8 (18.2%) | 26 (59.1%) | p=0.65 **^1^** |
|  |  | No detection | 11 (25.0%) | 7 (15.9%) | 18 (40.9%) | OR=0.7 |
|  |  | Sum | 29 (65.9%) | 15 (34.1%) | 44 (100%) | (0.3; 2.0) |
|  |  |  |  |  |  |  |
|  | Archaea | Detection | 15 (34.1%) | 9 (20.4%) | 24 (54.5%) | p=0.23 **^1^** |
|  |  | No detection | 16 (36.4%) | 4 (9.1%) | 20 (45.5%) | OR=0.6 |
|  |  | Sum | 31 (70.5%) | 13 (29.5%) | 44 (100%) | (0.2; 1.4) |
|  |  |  |  | |  |  |
| Males (N=44) | *P. gingivalis* | Detection | 13 (29.5%) | 20 (45.5%) | 33 (75.0%) | **p=0.004 ^1^** |
|  |  | No detection | 5 (11.4%) | 6 (13.6%) | 11 (25.0%) | **OR=4.0** |
|  |  | Sum | 18 (40.9%) | 26 (59.1%) | 44 (100%) | **(1.5; 13.6)** |
|  |  |  |  |  |  |  |
|  | *A. actinomycetemcomitans* | Detection | 9 (20.5%) | 13 (29.5%) | 22 (50.0%) | p=0.26 **^1^** |
|  |  | No detection | 7 (15.9%) | 15 (34.1%) | 22 (50.0%) | OR=1.9 |
|  |  | Sum | 16 (36.4%) | 28 (63.6%) | 44 (100%) | (0.7; 5.5) |
|  |  |  |  |  |  |  |
|  | *F. nucleatum* | Detection | 7 (15.9%) | 13 (29.6%) | 20 (45.5%) | p=0.84 **^1^** |
|  |  | No detection | 11 (25.0%) | 13 (29.5%) | 24 (54.5%) | OR=1.2 |
|  |  | Sum | 18 (40.9%) | 26 (59.1%) | 44 (100%) | (0.5; 2.9) |
|  |  |  |  |  |  |  |
|  | *S. sanguinis* | Detection | 9 (20.5%) | 14 (31.8%) | 23 (52.3%) | p=1.0 **^1^** |
|  |  | No detection | 14 (31.8%) | 7 (15.9%) | 21 (47.7%) | OR=1.0 |
|  |  | Sum | 23 (52.3%) | 21 (47.7%) | 44 (100%) | (0.4; 2.3) |
|  |  |  |  |  |  |  |
|  | Archaea | Detection | 8 (18.2%) | 8 (18.2%) | 16 (36.4%) | p=0.50 **^1^** |
|  |  | No detection | 12 (27.3%) | 16 (36.3%) | 28 (63.6%) | OR=0.7 |
|  |  | Sum | 20 (45.5%) | 24 (54.5%) | 44 (100%) | (0.2; 1.8) |

**^1^** Exact McNemar Test; 95% confidence interval (CI) for Odds Ratio (OR) is exact.

N, Number of matched pairs.

**Supporting Table S3.** Comparison of abundances between periodontally healthy (controls) and periodontally diseased subjects (cases).

| **Group** | **Taxon** | **Controls** | **Cases** | **P ^1^** |
| --- | --- | --- | --- | --- |
| 35-44 years  (N=43) | *P. gingivalis* | 0 (0; 9.7⋅10^-6^) | 9.5⋅10^-5^ (0; 5.2⋅10^-4^) | **<0.001** |
|  | *A. actinomycetemcomitans* | 6.0⋅10^-8^ (0; 1.4⋅10^-6^) | 0 (0; 1.7⋅10^-6^) | 0.42 |
|  | *F. nucleatum* | 0 (0; 6.5⋅10^-4^) | 2.5⋅10^-5^ (0; 3.0⋅10^-4^) | 0.70 |
|  | *S. sanguinis* | 1.6⋅10^-6^ (0; 2.8⋅10^-5^) | 5.8⋅10^-6^ (0; 5.8⋅10^-5^) | 0.19 |
|  | Archaea^2^ | 105.3 (0; 471.1) | 0 (0; 1966.4) | 0.70 |
|  | % Archaea^3^ | 0.0047 (0; 0.0176) | 0 (0; 0.0522) | 0.55 |
|  | Bacteria^12^ | 2.2 ⋅10^6^ (1.5⋅10^6^; 2.7⋅10^6^) | 2.6⋅10^6^ (1.9⋅10^6^; 3.6⋅10^6^) | 0.08 |
| 45-54 years  (N=45) | *P. gingivalis* | 3.9⋅10^-6^ (0; 4.0⋅10^-5^) | 1.7⋅10^-4^ (4.3⋅10^-5^; 9.2⋅10^-4^) | **<0.001** |
|  | *A. actinomycetemcomitans* | 1.9⋅10^-7^ (0; 3.9⋅10^-6^) | 1.5⋅10^-7^ (0; 5.6⋅10^-6^) | 0.85 |
|  | *F. nucleatum* | 0 (0; 9.3⋅10^-4^) | 2.1⋅10^-5^ (0; 1.0⋅10^-3^) | 0.36 |
|  | *S. sanguinis* | 1.2⋅10^-5^ (0; 1.2⋅10^-4^) | 9.7⋅10^-7^ (0; 2.7⋅10^-5^) | **0.007** |
|  | Archaea^2^ | 274.7 (0; 619.2) | 398.4 (0; 1.3⋅10^3^) | 0.58 |
|  | % Archaea^3^ | 0.0118 (0; 0.0340) | 0.0088 (0; 0.0380) | 0.76 |
|  | Bacteria^12^ | 2.0⋅10^6^ (1.5⋅10^6^; 2.6⋅10^6^) | 2.2⋅10^6^ (1.5⋅10^6^; 3.6⋅10^6^) | 0.11 |
| Females  (N=44) | *P. gingivalis* | 0 (0; 1.4⋅10^-5^) | 6.1⋅10^-5^ (0; 3.5⋅10^-4^) | **<0.001** |
|  | *A. actinomycetemcomitans* | 6.9⋅10^-7^ (0; 3.0⋅10^-6^) | 0 (0; 1.1⋅10^-6^) | **0.01** |
|  | *F. nucleatum* | 0 (0; 3.2⋅10^-4^) | 1.9⋅10^-4^ (0; 1.4⋅10^-3^) | **0.01** |
|  | *S. sanguinis* | 1.6⋅10^-5^ (0; 9.9⋅10^-5^) | 8.7⋅10^-6^ (0; 4.6⋅10^-5^) | 0.48 |
|  | Archaea^2^ | 246.8 (0; 554.1) | 296.3 (0; 1.4⋅10^3^) | 0.73 |
|  | % Archaea^3^ | 0.0120 (0; 0.0324) | 0.0088 (0; 0.0352) | 0.87 |
|  | Bacteria^12^ | 2.0⋅10^6^ (1.5⋅10^6^; 2.6⋅10^6^) | 2.5⋅10^6^ (1.7⋅10^6^; 3.6⋅10^6^) | 0.06 |
| Males  (N=44) | *P. gingivalis* | 0 (0; 3.2⋅10^-5^) | 1.7⋅10^-4^ (1.0⋅10^-5^; 9.4⋅10^-4^) | **<0.001** |
|  | *A. actinomycetemcomitans* | 0 (0; 1.4⋅10^-6^) | 6.4⋅10^-8^ (0; 9.5⋅10^-6^) | 0.12 |
|  | *F. nucleatum* | 0 (0; 1.1⋅10^-3^) | 0 (0; 1.9⋅10^-4^) | 0.27 |
|  | *S. sanguinis* | 5.0⋅10^-7^ (0; 3.7⋅10^-5^) | 6.3⋅10^-7^ (0: 2.3⋅10^-5^) | 0.38 |
|  | Archaea^2^ | 0 (0; 596.9) | 0 (0; 2063.4) | 0.44 |
|  | % Archaea^3^ | 0.0120 (0; 0.0324) | 0.0088 (0; 0.0352) | 0.87 |
|  | Bacteria^12^ | 2.1⋅10^6^ (1.5⋅10^6^; 2.9⋅10^6^) | 2.5⋅10^6^ (1.7⋅10^6^; 3.9⋅10^6^) | 0.12 |

Data are presented as median (25%; 75% quantile)

^1^ Wilcoxon matched-pairs signed-ranks test

^2^ proportion of 16S rRNA gene copies per ng extracted DNA; N, number of matched pairs.

^3^ percent of archaeal 16S rRNA gene copies per prokaryotic 16S rRNA gene copies (Archaea+Bacteria)*100

N, Number of matched pairs.

**Supporting Table S4**. Odds Ratios quantifying chance of being periodontally diseased depending on detection (yes/no) of different pathogens in tongue scrapings in subgroups according to age and gender.

| **Group** | **Taxon** | **OR (95% CI)** | **P value** |
| --- | --- | --- | --- |
| 35-44 years (N=43) | *P. gingivalis* | 3.73 (0.95; 14.55) | 0.06 |
|  | *A. actinomycetemcomitans* | 0.30 (0.08; 1.14) | 0.08 |
|  | *F. nucleatum* | 2.02 (0.53; 7.68) | 0.30 |
|  | *S. sanguinis* | 0.87 (0.28; 2.68) | 0.81 |
|  | *Archaea* | 0.34 (0.09; 1.32) | 0.12 |
| 45-54 years (N=45) | *P. gingivalis* | **4.68 (1.08; 20.27)** | **0.04** |
|  | *A. actinomycetemcomitans* | 0.48 (0.13; 1.76) | 0.27 |
|  | *F. nucleatum* | 1.34 (0.39; 4.59) | 0.64 |
|  | *S. sanguinis* | 0.31 (0.09; 1.12) | 0.07 |
|  | *Archaea* | 1.22 (0.38; 3.88) | 0.74 |
| Females (N=44) | *P. gingivalis* | **3.44 (1.00; 11.81)** | **0.05** |
|  | *A. actinomycetemcomitans* | **0.03 (0.002; 0.57)** | **0.02** |
|  | *F. nucleatum* | **5.58 (1.07; 28.99)** | **0.04** |
|  | *S. sanguinis* | 0.40 (0.12; 1.34) | 0.14 |
|  | *Archaea* | 0.51 (0.18; 1.49) | 0.22 |
| Males (N=44) | *P. gingivalis* | **10.42 (1.42; 76.78)** | **0.02** |
|  | *A. actinomycetemcomitans* | 1.46 (0.30; 7.16) | 0.64 |
|  | *F. nucleatum* | 0.66 (0.17; 2.51) | 0.54 |
|  | *S. sanguinis* | 1.01 (0.30; 3.46) | 0.99 |
|  | *Archaea* | 1.11 (0.30; 4.09) | 0.87 |

Conditional logistic regression modeling periodontal status (cases versus controls, dependent variable) on detection of different pathogens (yes/no) adjusting for age (cont.), school education, smoking status and BMI.

N, Number of matched pairs.

**Supporting Table S5.** Adjusted Odds Ratios quantifying chance of being periodontally diseased depending on **count**^◊^ different pathogens in tongue scrapings in the overall study population (N=88 pairs).

|  | **OR (95% CI)** | | |  |
| --- | --- | --- | --- | --- |
| **Taxon** | **T1** | **T2** | **T3** | **P for trend** |
| *P. gingivalis* | 1.00 | 1.60 (0.58; 4.44) | **14.05 (3.47; 56.87) ***** | **<0.001** |
| *A. actinomycetemcomitans* | 1.00 | 0.47 (0.15; 1.46) | **0.38 (0.15; 0.99) *** | **0.045** |
| *F. nucleatum* | 1.00 | 3.50 (0.74; 16.51) | 1.01 (0.42; 2.39) | 0.95 |
| *S. sanguinis* | 1.00 | 1.23 (0.47; 3.21) | 0.44 (0.17; 1.11) | 0.13 |
| Archaea | 1.00 | 0.45 (0.16; 1.26) | 1.02 (0.44; 2.36) | 0.99 |
| Bacteria | 1.00 | 0.62 (0.23; 1.69) | **0.09 (0.02; 0.36) **** | **0.001** |

Conditional logistic regression modeling periodontal status (cases versus controls, dependent variable) on single species, archaeal or bacterial count adjusting for age (cont.), school education, smoking status and BMI.

^◊^ Counts were categorized as tertiles (T1-T3). Numbers within tertiles were: *P. gingivalis*: 75-43-58, *A. actinomycetemcomitans*: 92-26-58, *F. nucleatum*: 93-25-58, *S. sanguinis*: 75-43-58, Archaea 85-33-58, Bacteria: 59-59-58.

* p<0.05, ** p<0.01, *** p<0.001 versus reference group
